# Association of Sleep and β-Amyloid Pathology Among Older Cognitively Unimpaired Adults

**DOI:** 10.1001/jamanetworkopen.2021.17573

**Published:** 2021-07-23

**Authors:** Philip S. Insel, Brian S. Mohlenhoff, Thomas C. Neylan, Andrew D. Krystal, R. Scott Mackin

**Affiliations:** 1 Department of Psychiatry and Behavioral Sciences, University of California, San Francisco; 2 Clinical Memory Research Unit, Faculty of Medicine, Lund University, Lund, Sweden; 3 Mental Health Service, Department of Veterans Affairs Medical Center, San Francisco, California

## Abstract

**Importance:**

Disrupted sleep commonly occurs with progressing neurodegenerative disease. Large, well-characterized neuroimaging studies of cognitively unimpaired adults are warranted to clarify the magnitude and onset of the association between sleep and emerging β-amyloid (Aβ) pathology.

**Objective:**

To evaluate the associations between daytime and nighttime sleep duration with regional Aβ pathology in older cognitively unimpaired adults.

**Design, Setting, and Participants:**

In this cross-sectional study, screening data were collected between April 1, 2014, and December 31, 2017, from healthy, cognitively unimpaired adults 65 to 85 years of age who underwent florbetapir F 18 positron emission tomography (PET), had *APOE* genotype information, scored between 25 and 30 on the Mini-Mental State Examination, and had a Clinical Dementia Rating of 0 for the Anti-Amyloid Treatment in Asymptomatic Alzheimer Disease (A4) Study. Data analysis was performed from December 1, 2019, to May 10, 2021.

**Exposures:**

Self-reported daytime and nighttime sleep duration.

**Main Outcomes and Measures:**

Regional Aβ pathology, measured by florbetapir PET standardized uptake value ratio.

**Results:**

Amyloid PET and sleep duration information was acquired on 4425 cognitively unimpaired participants (mean [SD] age, 71.3 [4.7] years; 2628 [59.4%] female; 1509 [34.1%] tested Aβ positive). Each additional hour of nighttime sleep was associated with a 0.005 reduction of global Aβ standardized uptake value ratio (*F*_1, 4419_ = 5.0; *P* = .03), a 0.009 reduction of medial orbitofrontal Aβ (*F*_1, 4419_ = 17.4; *P* < .001), and a 0.011 reduction of anterior cingulate Aβ (*F*_1, 4419_ = 15.9; *P* < .001). When restricting analyses to participants who tested Aβ negative, nighttime sleep was associated with a 0.006 reduction of medial orbitofrontal Aβ (*F*_1,2910_ = 16.9; *P* < .001) and a 0.005 reduction of anterior cingulate Aβ (*F*_1,2910_ = 7.6; *P* = .03). Daytime sleep was associated with a 0.013 increase of precuneus Aβ (*F*_1,2910_ = 7.3; *P* = .03) and a 0.024 increase of posterior cingulate Aβ (*F*_1,2910_ = 14.2; *P* = .001) in participants who tested Aβ negative.

**Conclusions and Relevance:**

In this cross-sectional study, the increased risk of Aβ deposition with reduced nighttime sleep duration occurred early, before cognitive impairment or significant Aβ deposition. Daytime sleep may be associated with an increase in risk for early Aβ accumulation and did not appear to be corrective for loss of nighttime sleep, demonstrating a circadian rhythm dependence of sleep in preventing Aβ accumulation. Treatments that improve sleep may reduce early Aβ accumulation and aid in delaying the onset of cognitive dysfunction associated with early Alzheimer disease.

## Key Points

**Question:**

What is the magnitude and time of onset of the association between daytime and nighttime sleep with β-amyloid (Aβ) pathology in cognitively unimpaired older adults?

**Findings:**

In this cross-sectional study of 4425 cognitively unimpaired participants, each additional hour of nighttime sleep was associated with a statistically significant reduction of Aβ positron emission tomographic standardized uptake value ratio, whereas daytime sleep was associated with increased regional accumulation of Aβ. The association occurs early, before significant Aβ accumulation or cognitive impairment, and in specific regions of the brain.

**Meaning:**

If longer sleep duration leads to reduced amyloid levels, treatments increasing sleep duration may reduce Aβ accumulation and aid in delaying the onset of cognitive dysfunction associated with Aβ deposition.

## Teaser

This cross-sectional study evaluates the associations between daytime and nighttime sleep duration with regional β-amyloid pathology in older cognitively unimpaired adults.

## Introduction

Sleep disruption has been proposed to play a role in increasing amyloid β (Aβ) deposition,^1,2,3^ the defining characteristic of the preclinical phase of Alzheimer disease, thought to begin decades before symptom onset.^4^ Increasing age and the ε4 allele of the *APOE* gene are principal risk factors for Aβ deposition. Cognitively unimpaired older adults with elevated levels of Aβ are at increased risk for cognitive decline during 3 to 6 years.^5^ Extracellular Aβ aggregation in mice increased with wakefulness, and humans exhibited waking-related increased levels of Aβ in cerebrospinal fluid (CSF).^6^ Chronic partial sleep restriction in rodents has also been experimentally found to increase Aβ deposition.^7^ Sleep deprivation is thought to reduce interstitial fluid volume to levels insufficient to clear Aβ.^8,9^ In humans, acute experimental sleep deprivation increased overnight CSF Aβ levels by 25% to 30% compared with levels in sleeping controls.^10^ A study^11^ of partial sleep deprivation revealed loss of slow wave sleep (SWS) associated with an acute increase in next-morning CSF Aβ. Positron emission tomography (PET)–determined Aβ burden in healthy older individuals was strongly associated with SWS.^1^ The primary role of SWS in Aβ turnover is thought to be related to higher flow in the brain glymphatic system during SWS.^8,12,13^

Such results suggest that less sleep over time may be associated with increased levels of Aβ deposition, but reports^14,15,16,17,18,19,20^ are mixed and have been limited by small sample sizes. An experiment that involved chronic partial sleep deprivation in humans found no correlation with CSF Aβ.^14^ Several studies with fewer than 100 older adult participants found correlations between self-reported total sleep time^15,16^ and nocturnal awakenings^17^ with PET Aβ deposition, but this finding has been inconsistent.^18,19,20^

We analyzed a sample of 4425 healthy, cognitively unimpaired, older adults with self-reported nighttime and daytime sleep duration, health information, and florbetapir F 18 PET imaging. The aims of these analyses were to evaluate whether self-reported sleep durations were associated with increased Aβ deposition, whether there was a specific regional pattern of deposition associated with sleep, and how early the association developed during Aβ accumulation. Additional factors affecting sleep, including caffeine and alcohol consumption, exercise, and symptoms of depression, were also assessed.^21,22,23^

## Methods

### Participants

Participants were screened for inclusion in the Anti-Amyloid Treatment in Asymptomatic Alzheimer Disease (A4) study^24,25,26^ from April 1, 2014, to December 31, 2017, across 67 sites in the US, Canada, Australia, and Japan. Members of the community were recruited using central media and local outreach initiatives. More than 15 000 individuals underwent prescreening via telephone or the A4 study website for initial minimal exclusionary criteria. Participants were included in this study if they were 65 to 85 years of age, were cognitively unimpaired, underwent a florbetapir PET scan, had *APOE* genotype information, scored 25 to 30 on the Mini-Mental State Examination Survey (MMSE), and had a Clinical Dementia Rating of 0. Data analysis was performed from December 1, 2019, to May 10, 2021. Informed written consent was obtained from all participants at each site. All data were deidentified. This study was approved by the institutional review boards of all the participating institutions. This study followed the Strengthening the Reporting of Observational Studies in Epidemiology (STROBE) reporting guideline for cross-sectional studies.

Participants were excluded from the A4 study if they were (1) taking a prescription Alzheimer medication (acetylcholinesterase inhibitor and/or memantine); (2) had a current serious or unstable illness, including cardiovascular, hepatic, renal, gastroenterologic, respiratory, endocrinologic, neurologic, psychiatric, immunologic, or hematologic disease or other conditions that could interfere with the study; (3) had a history within the last 5 years of a serious infectious disease that affected the brain (including neurosyphilis, meningitis, or encephalitis) or head trauma that resulted in protracted loss of consciousness; (4) had a history within the last 5 years of a primary or recurrent malignant disease, with the exception of resected cutaneous squamous cell carcinoma in situ, basal cell carcinoma, cervical carcinoma in situ, or in situ prostate cancer with normal prostate-specific antigen level after treatment; (5) had a known history of HIV, clinically significant multiple or severe drug allergies, or severe posttreatment hypersensitivity reactions, including but not limited to erythema multiforme major, linear IgA dermatosis, toxic epidermal necrolysis, or exfoliative dermatitis; (6) were at serious risk for suicide or had a history within the past 2 years of major depression or bipolar disorder; (7) had a history within the past 5 years of long-term alcohol or drug abuse or dependence; or (8) were residing in a skilled nursing facility or nursing home. Participants were not evaluated for obstructive sleep apnea. Participants in the A4 study who did not have evidence of elevated brain Aβ as determined by PET at screening were not randomized to treatment but were included in the current study.

### Florbetapir PET

Aβ PET was performed using florbetapir data, acquired 50 to 70 minutes after injection. Images were realigned and averaged and then spatially aligned to a standard space template. Florbetapir, sampled in a global neocortical region for Aβ (an average of the anterior and posterior cingulate, precuneus, medial orbitofrontal, temporal and parietal lobes), was expressed as a standardized uptake value ratio (SUVR) with a cerebellar reference region.^27^ A previously identified threshold was used to define Aβ positivity (florbetapir PET SUVR≥1.10).^28,29^ The individual regions of interest were also evaluated.

### Sleep

Recent mean hours of nighttime sleep was measured via participant self-report during the screening visit, with responses including integer values ranging from 2 to 12. Participants were asked about their “average total number of hours slept at night” and “average total number of minutes napped during the day.” Three participants reported fewer than 4 hours of nightly sleep, and 5 participants reported greater than 10 hours of nightly sleep. For the analyses, the sleep distribution was truncated at 4 and 10 hours of sleep. Recent “average number of minutes of daytime sleep” was also self-reported, ranging from 0 to 240 minutes.

### Statistical Analysis

The goal of this analysis was to evaluate the association between Aβ PET SUVR (as a continuous measure and dichotomized as Aβ positive or negative) and self-reported duration of nighttime and daytime sleep. Global Aβ PET SUVR as well as regional Aβ were regressed on mean hours of sleep using robust linear regression.^30^ A potential nonlinear association between hours of sleep and Aβ PET SUVR was evaluated using restricted cubic splines. The association between Aβ PET SUVR and sleep was tested using robust *F* tests. We also tested for interactions between hours of nighttime sleep and age, sex and *APOE* genotype (ε4^+^ vs ε4^−^), each separately, to predict Aβ PET SUVR.

Models with Aβ positivity as the outcome were fit with logistic regression. Models that predicted Aβ PET SUVR and Aβ positivity included age, sex, *APOE* genotype (ε4^+^ vs ε4^−^) as well as caffeine and alcohol consumption, exercise, and symptoms of depression. Estimates of nighttime sleep were adjusted for daytime sleep, and estimates of daytime sleep were adjusted for nighttime sleep, both through covariate adjustment.

For regions where Aβ PET SUVR was significantly associated with nighttime sleep duration, a joint mixed-effects model with a random intercept was used to test whether the regional association was different from the association between global Aβ PET SUVR and nighttime sleep. Vectors of regional and global SUVR responses were concatenated^31^ and predicted by the interaction between region and nighttime sleep duration, adjusting for age, sex, *APOE* genotype, and daytime sleep.

To assess whether the association between sleep duration and Aβ occurred early in the accumulation process, before significant Aβ deposition, all analyses were repeated, restricting to participants who tested Aβ negative (SUVR < 1.10).

Associations between sleep and cohort characteristics (age, sex, educational level, *APOE* genotype, and MMSE) as well as daily habits (daily number of cups of caffeine consumed, daily number of alcoholic drinks consumed, weekly number of hours of aerobic exercise, and daily number of minutes spent walking) were assessed using a Kruskal-Wallis test for categorical variables and Spearman correlation for continuous variables. Symptoms of depression, measured by the Geriatric Depression Scale (GDS), were also evaluated. The GDS scores were categorized into 3 groups (0, no depression; 1-5, subsyndromal symptoms of depression; and 6, major depressive symptoms). A 2-sided *P* < .05 was considered significant. Regional Aβ PET analyses were corrected for multiple comparisons using a Holm adjustment.^32^ All analyses were performed in R, version 4.0.2 (R Foundation for Statistical Computing). Brain maps were created using fsbrain.^33^

## Results

### Cohort Characteristics

Amyloid PET and sleep duration information was acquired on 4425 cognitively unimpaired participants (mean [SD] age, 71.3 [4.7] years; 2628 [59.4%] female). All participants had Clinical Dementia Rating scores of 0 and a mean MMSE score of 28.8 (range, 25-30). Participants had a mean (SD) of 16.6 (2.8) years of education, 1546 (34.9%) tested *APOE* ε4 positive, and 1509 (34.1%) tested Aβ positive. The mean (SD) Aβ PET SUVR was 1.09 (0.19). The mean (SD) hours of nighttime sleep was 7.1 (1.1), with 2957 participants (66.8%) reporting 7 to 8 hours of sleep, 283 (6.4%) reporting greater than 8 hours of sleep, and 1185 (26.8%) reporting 6 or fewer hours of sleep. A total of 2745 participants (62.0%) reported no daytime sleep. Among daytime sleepers, the mean (SD) duration of sleep was 33.0 (25.7) minutes.

Nighttime sleep duration was not associated with age, *APOE* ε4 status, caffeine consumption, or exercise (Table). More nighttime sleep was associated with female sex, more education, more alcohol consumption, less daytime sleep, fewer symptoms of depression, and higher MMSE score (Table).

**Table.** Cohort Characteristics and Associations With Sleep Duration

Characteristic	Finding^a^	ρ^b^	*P* value
Age	71.3 (4.7) [65-85]	−0.002	.88
Sex, No. (%)			
Male	1797 (40.6)	7.07	.02
Female	2628 (59.4)	7.13	
Educational level, y	16.6 (2.8) [7-32]	0.05	.001
*APOE*, No. (%)			
ε4 Negative	2879 (65.1)	7.10	.47
ε4 Positive	1546 (34.9)	7.12	
MMSE score	28.8 (1.2) [25-30]	0.05	.001
Daytime sleep, min/d	12.5 (22.5) [0-240]	−0.04	.01
Caffeine, c/d	2.2 (1.9) [0-12]	0.02	.32
Aerobic exercise, h/wk	2.8 (3.6) [0-20]	0.01	.46
Walking, min/d	58.7 (61.3) [0-400]	−0.001	.94
Alcohol, drinks/d	0.8 (1.0) [0-5]	0.06	<.001
GDS score			
0	2121 (47.9) [0]	7.14	.004
1-5	2215 (50.1) [1-5]	7.08	
≥6	89 (2.0) [6-13]	6.81	

Abbreviation: GDS, Geriatric Depression Scale; MMSE, Mini-Mental State Examination.

^a^ Data are reported as mean (SD) [range] unless otherwise indicated.

^b^ Correlation with hours of sleep (or group means).

### Sleep and Aβ PET

**Figure 1.**
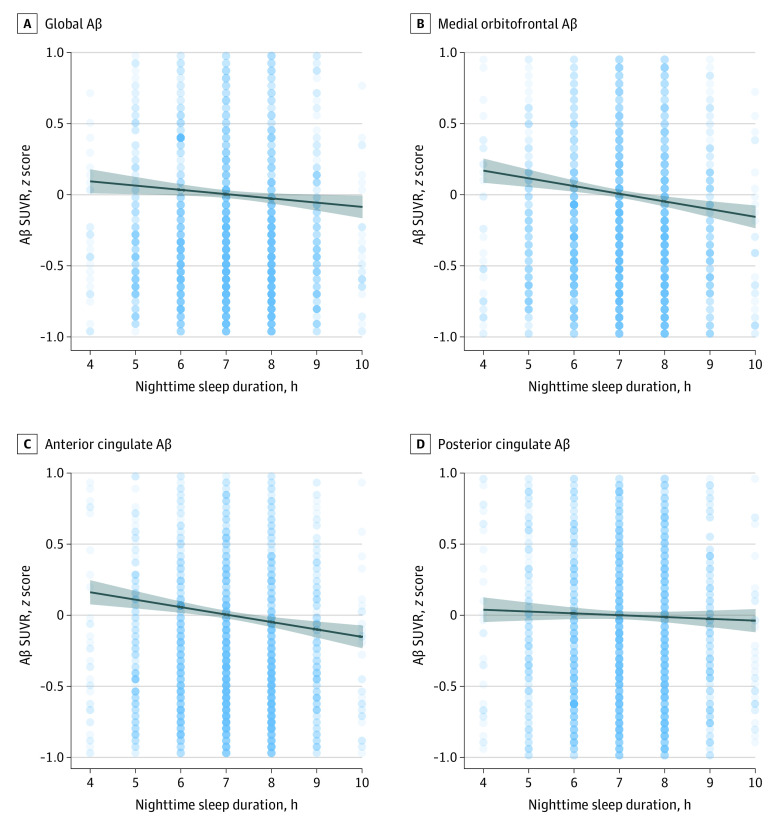
Duration of Nighttime Sleep and β-Amyloid (Aβ) Pathology Global Aβ positron emission tomographic standardized uptake value ratio (SUVR) and 3 regions of interest are plotted against hours of nighttime sleep. The center curve indicates the population curve, and the shaded areas indicate 95% CIs. The SUVRs are *z* score transformed across all participants.

**Figure 2.**
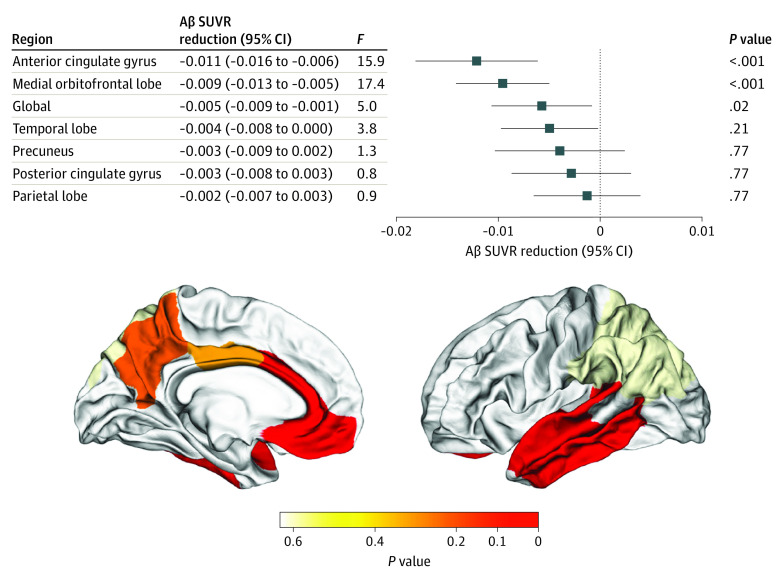
Nighttime Sleep and Estimates of β-Amyloid (Aβ) Reduction Estimates of Aβ reduction and corresponding CIs (error bars) are shown for each region of interest for every hour of increased duration of nighttime sleep. Dashed line indicates no reduction in Aβ. SUVR indicates standardized uptake value ratio.

After adjustment for age, sex, *APOE* genotype, and daytime sleep duration, each additional hour of nighttime sleep was associated with a 0.005 reduction of global Aβ PET SUVR (*F*_1,4419_ = 5.03; *P* = .02) (Figure 1A-D). Nighttime sleep was associated with a 0.009 reduction of medial orbitofrontal Aβ (*F*_1,4419_ = 17.4; *P* < .001) and a 0.011 reduction of anterior cingulate Aβ (*F*_1,4419_ = 15.9; *P* < .001) for each additional hour of nighttime sleep (Figure 1 and Figure 2). Regional Aβ estimates for nighttime sleep, adjusting for age, sex, *APOE* genotype, and daytime sleep duration, are summarized in Figure 2.

After additional adjustments for alcohol and caffeine consumption, exercise and symptoms of depression, the estimates were similar, with each additional hour of nighttime sleep associated with a 0.005 reduction of global Aβ PET SUVR (*F*_1,4406_ = 4.91; *P* = .03). After the additional adjustments, nighttime sleep was associated with a 0.009 reduction of medial orbitofrontal Aβ (*F*_1,4406_ = 17.2; *P* < .001) and a 0.011 reduction of anterior cingulate Aβ (*F*_1,4406_ = 15.3; *P* < .001) for each additional hour of nighttime sleep.

The difference in the association between nighttime sleep and regional Aβ PET SUVR from the association between nighttime sleep and global Aβ PET SUVR was also tested. The reduction in anterior cingulate Aβ for each additional hour of nighttime sleep was significantly greater than the reduction in global Aβ (β = −0.006; *P* = .001). The reduction in medial orbitofrontal Aβ for each additional hour of nighttime sleep was greater than the reduction in global Aβ (β = −0.004; *P* = .054).

Each additional hour of nighttime sleep was associated with a 6% reduction in the risk of Aβ positivity (log odds ratio: −0.064; *P* = .048). After adjusting for alcohol, caffeine, exercise, and symptoms of depression, the finding was not significant (log odds ratio: −0.063; *P* = .052). The model for the association between nighttime sleep duration and Aβ PET SUVR was not significantly improved by a nonlinear parameterization (global SUVR: χ^2^ = 0.54; *P* = .46; anterior cingulate: χ^2^ = 1.64; *P* = .20; posterior cingulate: χ^2^ = 0.02; *P* = .89; parietal lobe: χ^2^ = 0.25; *P* = .62; precuneus: χ^2^ = 0.02; *P* = .87; temporal lobe: χ^2^ = 2.11; *P* = .15; and medial orbitofrontal: χ^2^ = 0.23; *P* = .63). Daytime sleep duration was not associated with global Aβ (β = −0.002; *F*_1,4419_ = 0.15; *P* = .70) or regional Aβ (anterior cingulate: β = −0.010; F_1,4419_ = 1.51; *P* = .66; posterior cingulate: β = 0.012; F_1,4419_ = 2.06; *P* = .61; parietal lobe: β = −0.003; F_1,4419_ = 0.18; *P* = .67; precuneus: β = 0.008; F_1,4419_ = 1.09; *P* = .66; temporal lobe: β = −0.013; F_1,4419_ = 5.12; *P* = .14; medial orbitofrontal: β = −0.012; F_1,4419_ = 3.68; *P* = .28).

The association between nighttime sleep duration and Aβ PET SUVR did not differ by age (*F*_1,4418_ = 0.02; *P* = .89), sex (*F*_1,4418_ = 0.83; *P* = .36), or *APOE* status (*F*_1,4418_ = 1.12; *P* = .29).

### Sleep and Aβ PET Within Participants Who Tested Aβ Negative

**Figure 3.**
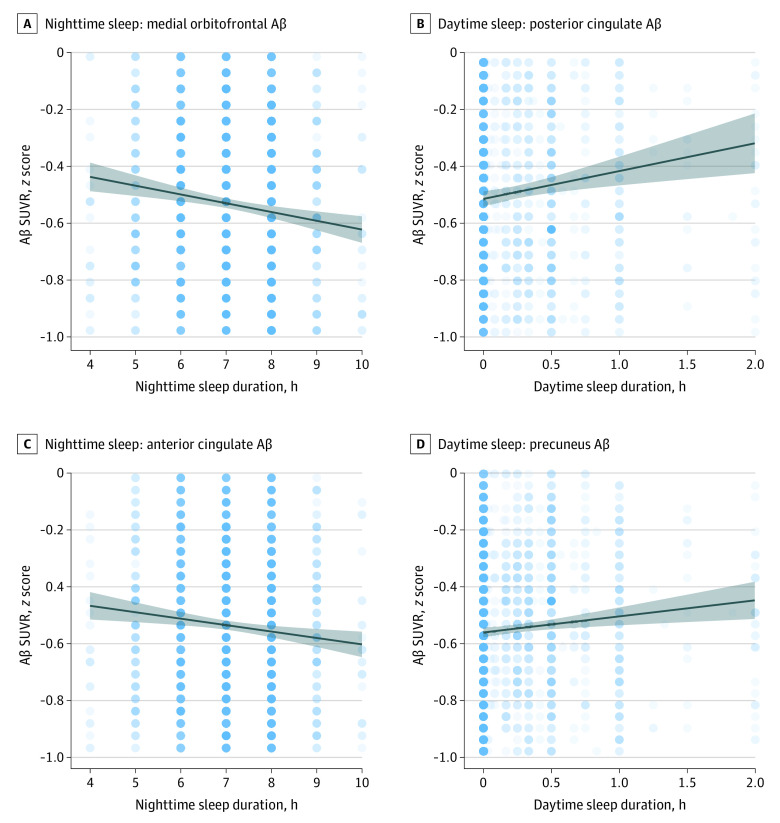
Duration of Nighttime and Daytime Sleep and β-Amyloid (Aβ) Pathology in Participants Who Tested Aβ Negative Regional Aβ positron emission tomographic standardized uptake value ratio (SUVR) is plotted against nighttime sleep duration in the column on the left and daytime sleep duration in the column on the right for participants who tested Aβ negative. The center curve indicates population curve, and the shaded areas indicate 95% CIs. The SUVRs are z score transformed across all participants, but only participants who tested Aβ negative were included in these analyses.

To assess whether the association between sleep and Aβ accumulation occurs in the early stages of accumulation, before significant Aβ deposition, analyses were performed again in 2916 participants with Aβ levels in the normal range (SUVR < 1.10). Nighttime sleep was not associated with global Aβ PET SUVR in participants who tested Aβ negative (β = −0.001; *F*_1,2910_ = 0.69; *P* = .41). Nighttime sleep was associated with a 0.006 reduction of medial orbitofrontal Aβ (*F*_1,2910_ = 16.9; *P* < .001) and a 0.005 reduction of anterior cingulate Aβ (*F*_1,2910_ = 7.6; *P* = .03) for each additional hour of nighttime sleep (Figure 3). Nighttime sleep was not associated with Aβ in any other region (posterior cingulate: β = 0.003; F_1,2910_ = 1.70; *P* = .76; parietal lobe: β = −0.0001; F_1,2910_ = 0.00; *P* > .99; precuneus: β = 0.002; F_1,2910_ = 1.71; *P* = .76; temporal lobe: β = −0.001; F_1,2910_ = 0.44; *P* > .99).

Daytime sleep duration was not associated with global Aβ in participants who tested Aβ negative (β = 0.005; *F*_1,2910_ = 2.0; *P* = .16). Daytime sleep was significantly associated with a 0.024 increase of posterior cingulate Aβ (*F*_1,2910_ = 14.2; *P* = .001) and a 0.013 increase of precuneus Aβ (*F*_1,2910_ = 7.3; *P* = .03) for each additional hour of daytime sleep (Figure 3). Daytime sleep was not associated with Aβ in any other region (anterior cingulate: β = 0.0006; F_1,2910_ = 0.02; *P* > .99; parietal lobe: β = 0.004; F_1,2910_ = 0.56; *P* > .99; temporal lobe: β = −0.007; F_1,2910_ = 3.36; *P* = .27; medial orbitofrontal lobe: β = −0.002; F_1,2910_ = 0.38; *P* > .99). No change occurred in the association between nighttime or daytime sleep duration and Aβ PET SUVR in the participants who tested Aβ negative when adjusting for alcohol and caffeine consumption, exercise, and depression.

## Discussion

These analyses suggest that healthy, cognitively unimpaired, older adults who reported longer total nighttime sleep had lower levels of Aβ brain deposition. In addition, the sleep-Aβ association was already apparent in early stages of Aβ accumulation in participants without abnormally elevated levels of Aβ. Nighttime sleep duration had a protective association against early Aβ accumulation, whereas daytime sleep was associated with increased deposition in regions known to be among the earliest sites of accumulation in the Aβ cascade.^34^

The large sample size in this study (N = 4425) provides precise estimates of the association between Aβ pathology and self-reported sleep duration, which to date has been observed inconsistently in smaller studies.^15,16,17,18,19,20,21,22^ Notably, these data did not suggest that moderate amounts of sleep might be better than longer durations in terms of reduced Aβ burden, unlike the quadratic shape that captures the association between sleep duration and cognition.^35^ No apparent ceiling effect was found for the association between sleep and Aβ; the association between longer nighttime sleep duration up to 10 hours and lower Aβ burden extended to the longest sleep durations, as demonstrated by the linear association between sleep duration and Aβ burden. These findings support the hypothesis that sleep facilitates clearance of Aβ from the human brain.^16^ It is also possible that Aβ itself disrupts sleep^1^ or that a third unobserved process affects sleep and Aβ deposition.

Although excessive daytime sleepiness has been reported to be associated with increased levels of Aβ,^36,37^ daytime sleep itself has not previously been reported to be associated with Aβ burden,^36^ and the association has not been demonstrated to already be present in individuals without abnormal levels of Aβ or cognitive impairment. Higher Aβ levels were associated with increasing daytime sleep in the posterior cingulate and precuneus, both high accumulation rate regions in Aβ-negative individuals.^34,38^ Excessive daytime sleepiness at baseline reportedly predicts subsequent Aβ accumulation in the posterior cingulate, precuneus as well as the anterior cingulate in elderly people with mixed Aβ pathology and no dementia.^37^ These results also align with the decreased levels of Aβ in the anterior cingulate and medial orbitofrontal lobe associated with increased nighttime sleep duration observed here. The finding that daytime sleep was not associated with Aβ in the full cohort suggests that the association between daytime sleep and Aβ may change with increasing levels of Aβ pathology. Heterogeneity of responses frequently increases with advancing pathology, and the variation in sleep patterns in response to elevated levels of Aβ may increase. Still, in both the full cohort and the analysis of the Aβ-negative group alone, daytime sleep was not associated with lower levels of Aβ, suggesting that there may be a circadian rhythm dependence to the protective association of sleep with Aβ accumulation. This finding also suggests that one may not be able to make up for the Aβ deposition associated with nighttime sleep deficits with daytime sleep.

The magnitude of Aβ reduction with additional nighttime sleep or increase with daytime sleep varied by site of deposition. The largest estimate was a 0.024 SUVR increase in the posterior cingulate associated with an hour of daytime sleep in the Aβ-negative group. A 0.024 SUVR difference corresponds to 0.13 SDs of the global Aβ signal, which is a relatively small effect size. The estimates of Aβ SUVR reduction in the anterior cingulate and medial orbitofrontal lobe of 0.01 and 0.005 in global Aβ are smaller still; however, these estimates are per hour of additional nighttime sleep increase; the difference between getting 6 vs 9 hours of sleep per night starts to represent a substantial effect size. It is unknown during what period the sleep Aβ association evolved. Longitudinal follow-up will be required for such temporal estimates and to further evaluate the clinical meaningfulness of these effect sizes.

The anterior and posterior dissociation of nighttime vs daytime sleep associations with Aβ PET burden was unexpected. The negative association of Aβ PET SUVR in the anterior cingulate and frontal regions may reflect clearance of emerging Aβ accumulation by nighttime sleep-related mechanisms; however, the lack of a similar protective association in the posterior cingulate or precuneus is puzzling, unless daytime napping is driven by a separate mechanism with regional specificity.

Information regarding sleep duration in this study was limited to self-report. The underestimate of self-reported sleep, compared with polysomnography, is strongly correlated with alterations in electroencephalographic frequency content during non–rapid eye movement sleep, suggesting that the sleep was physiologically lighter.^39^ This finding supports the notion that self-reported sleep duration, while providing different information from polysomnography, is valid in its own right as an indicator of the sleep experience. Moreover, the range of reported sleep durations (mostly 5-9 hours per night) was consistent with recent reports^35,40^ in older adults. The negative correlation observed in this study between nighttime sleep duration and Aβ deposition has also been observed using an objective measure of sleep.^41^ Finally, self-reported sleep duration is not without value clinically^42^ because these data suggest that self-reported sleep duration might be used to help predict brain amyloid burden. If longer nighttime sleep duration leads to reduced amyloid levels, treatments increasing nighttime sleep duration may reduce Aβ accumulation and aid in delaying the onset of cognitive dysfunction associated with Aβ deposition. Such treatments may be of particular value if they are administered early and slow Aβ accumulation before significant Aβ levels are deposited. However, still unknown is whether a protective effect will occur in terms of Aβ accumulation when sleep duration is increased with medications, which may depend on specific mechanisms of action. Although the association between sleep duration and Aβ burden was not altered by age, sex, or *APOE* status, attention to sleep duration may be important for those at higher risk for preclinical Alzheimer disease.

### Limitations

This study has some limitations. One limitation is that participants were not specifically screened for obstructive sleep apnea. Apart from other subjective or objective sleep parameters, untreated obstructive sleep apnea has been linked to brain Aβ deposition.^43^ However, because individuals with obstructive sleep apnea tend to underestimate their total sleep time more than individuals without obstructive sleep apnea,^44^ the effect of obstructive sleep apnea on brain Aβ deposition could be expected to inflate any observed correlation between sleep duration and amyloid deposition. Potential protective effects of sleep might be attenuated by the presence of untreated sleep apnea. Inflation of the observed association attributable to untreated obstructive sleep apnea in this study is possible, although participants reporting significant medical illnesses were excluded.

## Conclusions

Dementia associated with Alzheimer disease has been linked to amyloid deposits and altered sleep patterns^1^; however, it remains unknown whether sleep is a modifiable risk factor for Alzheimer disease or whether the observed association between greater Aβ deposition and shorter nighttime sleep duration or daytime sleep duration is attributable to a third, unobserved process. Regarding the latter, recent data indicate that Alzheimer disease is associated with marked degeneration of wake-regulating nuclei, which could account for increased daytime sleep.^45^ These results demonstrate that the association between sleep duration and Aβ burden occurs early, before cognitive impairment or significant Aβ deposition. Although these results add to the increasing evidence in support of an association between Aβ deposition and sleep duration, longitudinal information regarding sleep duration and change in Aβ deposition as well additional indicators of disease, such as cognitive functioning, will further clarify the role of sleep in amyloid accumulation. Future studies of sleep and Aβ accumulation may benefit from focusing on specific sites of deposition, including the orbitofrontal and cingulate cortices. Therapies aimed at improving sleep may be a viable strategy to slow early Aβ accumulation and subsequent cognitive dysfunction in the progression of Alzheimer disease.
